# High-efficiency dual-polarized broadband reflecting metasurface using continuous polarization conversion technique and element with multi degree of freedom

**DOI:** 10.1038/s41598-022-11694-8

**Published:** 2022-05-09

**Authors:** Majid Karimipour, Iman Aryanian

**Affiliations:** 1 Department of Electrical Engineering, Arak University of Technology, Arak, Iran; 2 Iran Telecommunication Research Center (ITRC), Tehran, Iran

**Keywords:** Electrical and electronic engineering, Design, synthesis and processing

## Abstract

In this study, two effective approaches are combined which are implemented at the element design level and system design level to simultaneously improve the frequency bandwidth and aperture efficiency of a dual-polarized single-layer reflecting metasurface. At the element design level, a broadband behavior is realized by using the polarization conversion technique (PCT) which is a novel technique to enhance the bandwidth of the element. To this end, an anisotropic metasurface with the I-shaped metal patch is proposed for rotating the polarization of the wave emitted from a point source by 90$^{\circ }$ and making a continuous phase shift in a full range of 360$^{\circ }$ within 8-18 GHz. Therefore, a completely equiphase aperture is achieved leading to enhancing the metasurface performance such as directivity and aperture efficiency and reducing the sidelobe level compared to reflecting metasurface developed by 1-bit phase quantization technique. At the system design level, the three-frequency phase synthesis (TFPS) method, which is based on determining the best constant reference phase for the aperture, is used and the corresponding constant reference phases are optimized to minimize the phase error in the whole band. The combination of TFPS and PCT enhances the effectiveness of the TFPS method considerably. An 841 element reflecting metasurface with aperture dimensions of 290 cm $\times$ 290 cm is designed, simulated, and fabricated in Ku-band to verify the concept. The measurement results show that the 1-dB gain bandwidth before and after combining PCT and TFPS techniques are 17.47% (14.1–16.8 GHz) and 30.3% (14–19 GHz), respectively. In addition, the maximum aperture efficiency of the proposed metasurface is 63.62% which occurs at 14.5 GHz.

## Introduction

Reflecting metasurface (or reflectarray) is a proper candidate for high gain applications such as satellite communication. The configuration of reflective metasurface can be artfully designed to improve wireless communication and power transfer^1,2^. This is why it benefits from the advantages of reflector and classic array structures^3^. In general, a reflecting metasurface manipulates the phase of the wave impinging from the feed and reflects it with the desired characteristic such as direction, shape, polarization, and beamwidth^4–7^. This is performed by introducing a given progressive phase shift over the reflected wave by designing a proper phase-shifting surface or making an artificial impedance surface to engineer the reflected waves^8^. Although the reflecting metasurface has numerous advantages, it suffers from narrowband behavior. Frequency bandwidth enhancement has always attracted much attention from communication engineers. So far, several efforts have been made to broaden the bandwidth of reflecting metasurface. These methods can be divided into two categories: (I) broadening approaches at the element design level, and (II) broadening approaches at the system design level.

Broadband methods at the system design level refer to those techniques that deal with the metasurface configuration neglecting the element type, such as phase synthesis methods. For instance, the authors in Ref.^9^ introduced a broadband method at the system design level based on double frequency phase shift (DFPS) synthesis and adopted an optimization routine to find the optimum constant reference phase at two-interval frequencies of the working band. It can be simply inferred that the DFPS technique has great potential to enhance the bandwidth neglecting the element type. For instance, a 16.7% of 1.5-dB gain bandwidth was achieved by a simple phasing element with a single-layered configuration^9^. Therefore, the effectiveness of the phase synthesis method can be considerably enhanced by using wideband phasing elements. Exploiting this fact, a broadband reflecting metasurface with a 1-dB gain bandwidth of 21% was developed^10^.

**Figure 1:**
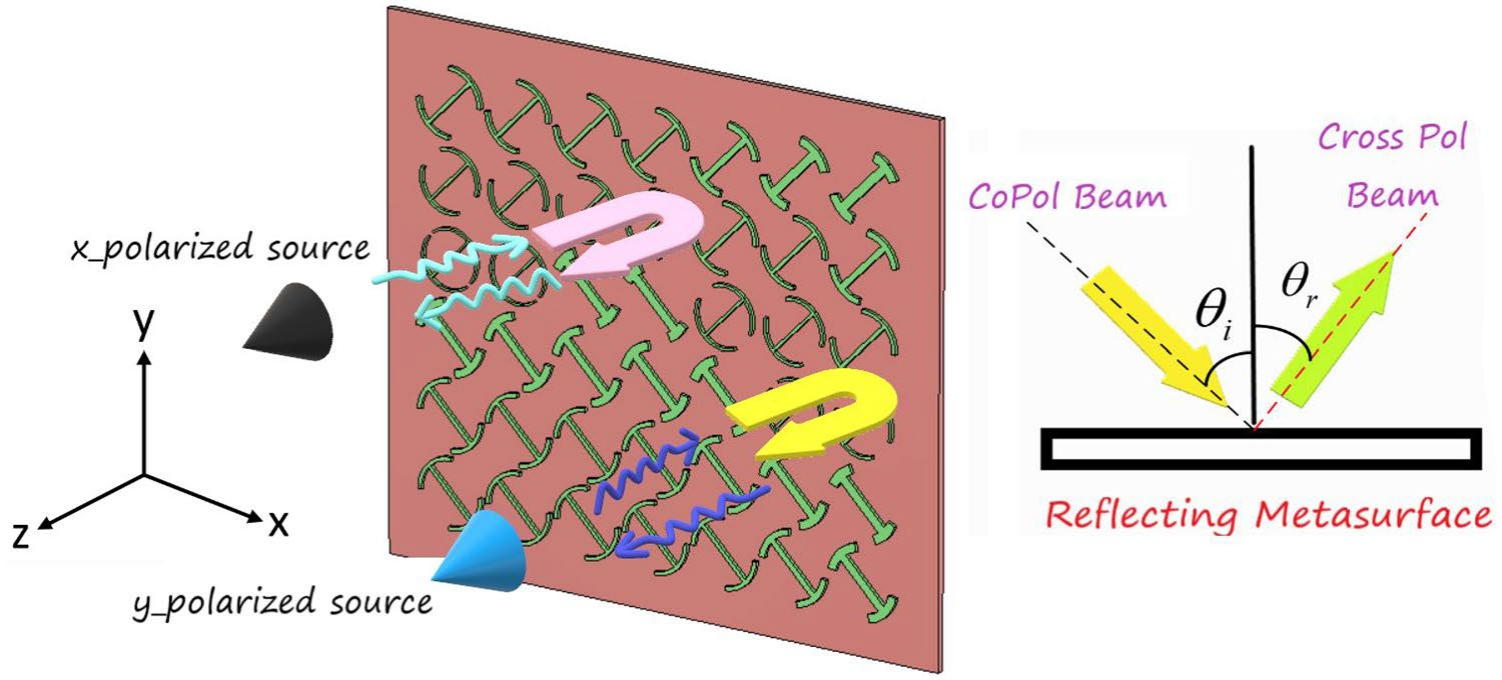
The proposed wideband reflecting metasurface working in the cross-polarization mode when illuminated by a point source.

At the element design level, several broadband techniques are introduced in the literature to improve the bandwidth behavior of phasing elements in reflecting metasurfaces^11–18^. Recently, a novel broadband technique has been proposed based on the polarization rotation of the impinging waves from the feed which makes the metasurface work in the cross-polarization mode^19^. This technique, along with the studies that followed (Ref.^20^ and Ref.^21^) improves the reflecting metasurface bandwidth up to 20% merely by utilizing single-layered configurations. In this regard, several works have been reported to design wideband or multi-band polarization conversion metasurfaces^22–26^. It can be understood that there is more freedom to manipulate the phase of reflected waves in the cross-polarization mode compared with the co-polarization (copol) one in reflective structures^19,21,27^. For example, the authors in Ref.^27^ exploited this interesting feature to design an ultra-thin transmit-reflecting metasurface structure. Similarly, the authors in Ref.^19^ developed a 1-bit geometrical spatial phase shifter to rotate the polarization of the reflected wave. Although the 1-bit reflecting metasurface has a simple architecture compared with a completely equiphase aperture, the radiation performance such as the aperture efficiency of the radiator is relatively poor in these structures. For instance, it can be inferred from Ref.^19^ that the imperfect beam collimation in 1-bit reflecting metasurface causes to 3.8 dB gain reduction in the pattern compared with the beams constructed by continuous phasing aperture. Furthermore, sidelobe level enhancement is inevitable in 1-bit phase quantization apertures specifically in the off-centered feed configuration. To circumvent this drawback, a polarization conversion unit cell that can generate continuous phase shifting over the reflected wave was introduced in which a 1-dB gain bandwidth up to 18.5% was reported using this technique^21^.

To further improve the bandwidth of reflecting metasurfaces and achieve high aperture efficiency simultaneously, two broadening approaches introduced in Ref.^9^ and Ref.^19^ are effectively combined and modified in this paper. As stated before, these two approaches are implemented at the element design and system design levels. At the element design level, a single layer I-shape element with its mirror counterpart is employed to not only cover a full cycle of 360$^{\circ }$ but also show an identical behavior in the reflection phase for the cross-polarization mode within 8–21 GHz. This goal is realized by adopting the multi-degree-of-freedom (MDoF) technology in the design of the element. Thanks to the use of lossless metasurface with reciprocal elements in the structure, time-reversal symmetry is maintained, which ensures that the metasurface displays a similar response for both y- and x-polarized impinging waves, and accordingly co and cross-polarization reflection coefficients are similar for both cases. Figure 1 depicts the working mechanism of the proposed reflecting metasurface in cross-polarization mode. Note that combining the method introduced in^9^ and^19^ confers a great advantage because the effectiveness of the broadband technique introduced in Ref.^19^ can further be improved if a broadband unit cell is utilized at the element design level.

**Figure 2:**
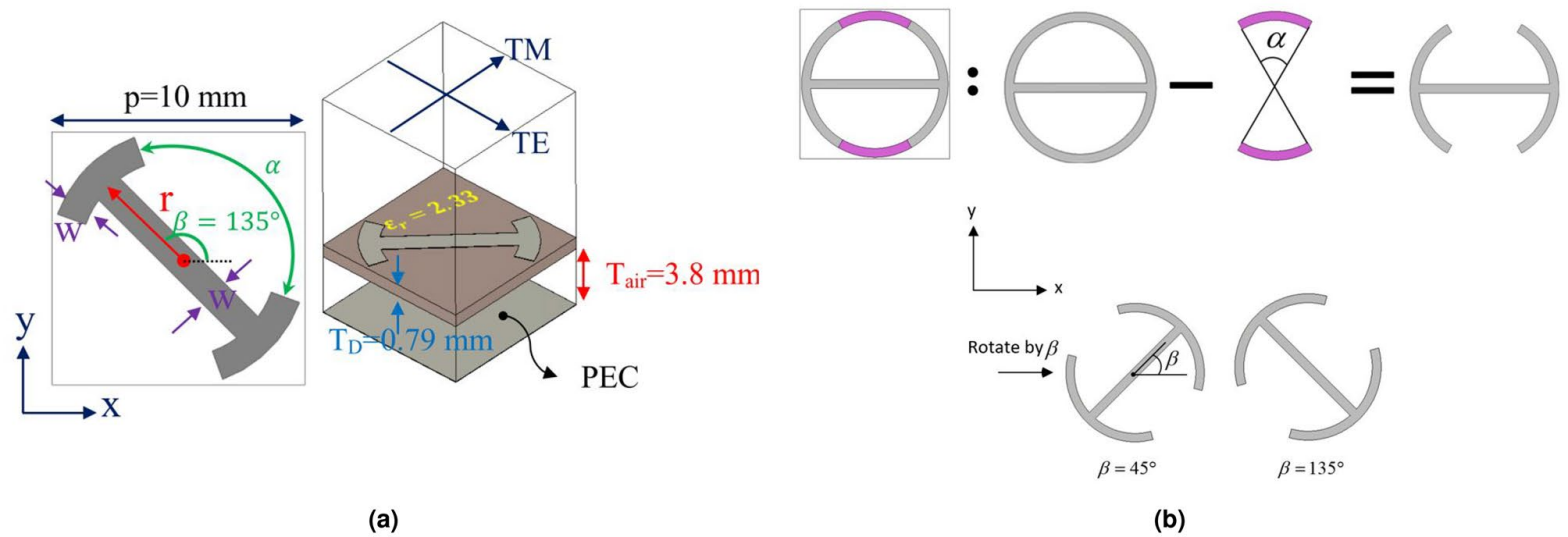
(**a**) Polarization rotating element and its geometrical parameters.(**b**) Construction steps of the metal patch of the unit cell element.

## Element design for polarization conversion

To control the electromagnetic characteristics of the reflected cross-polarization wave from the metasurface in a wide frequency range to generate a versatile radiation pattern and emulate the behavior of classical antennas, a continuous phasing aperture should be developed to show similar behavior in the frequency band of interest. The aim is to design an element working in the cross-polarization mode and covering the Ku frequency band. Theoretical investigations indicate that the main feature of a reflective linear polarization converter is that it reflects two x- and y-polarized electric fields with the same amplitude and ${180^ \circ }$ phase difference with respect to the illuminated wave components. Apparently, it can be deduced that the metasurface should be selected from anisotropic structures with negligible loss to keep the amplitude of the reflected wave equal to the illuminated one. The working principle of the linear polarization conversion can be interpreted by introducing an additional coordinate system with two perpendicular axes which are rotated by ${45^ \circ }$ with respect to the XY coordinate system. A detailed discussion of the theory is presented in Ref.^26^.

**Figure 3:**
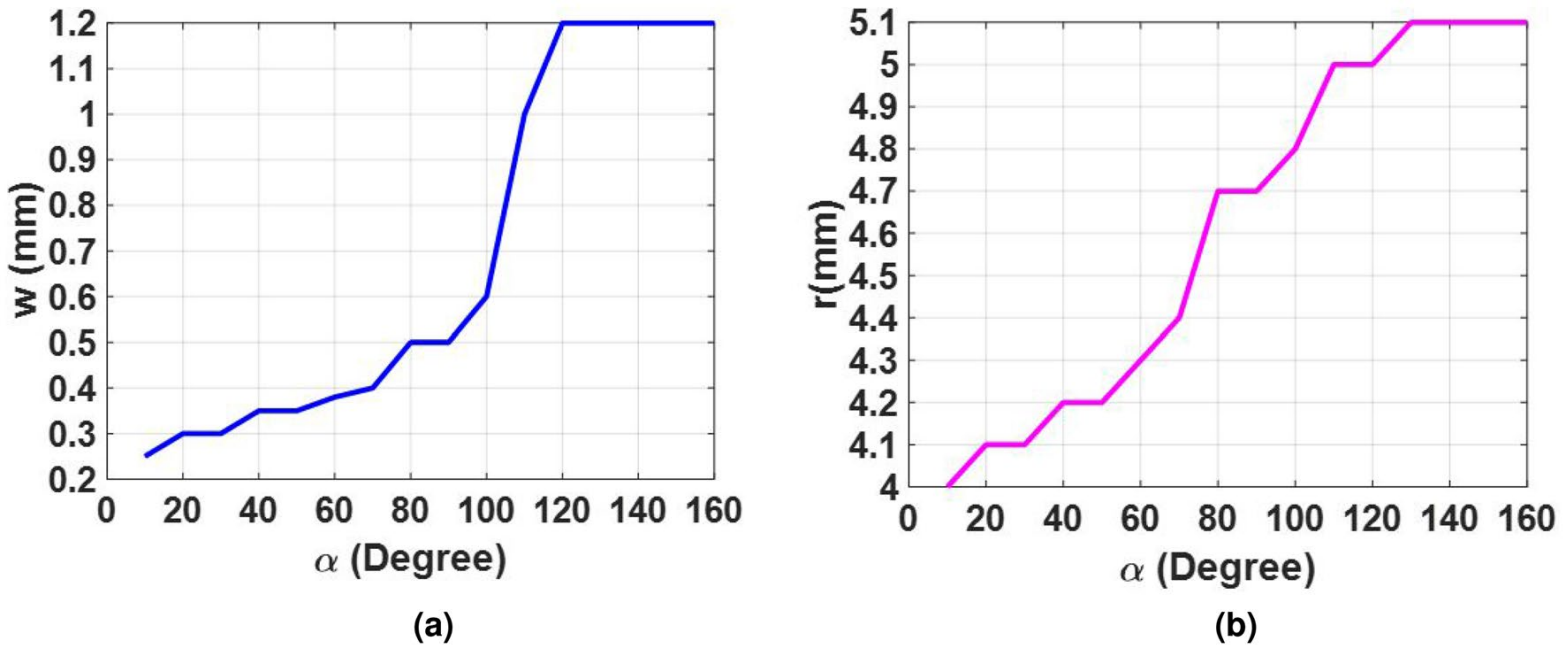
Optimum relation between the design parameters of the unit cell for realizing the wideband behavior in the cross-polarization mode. (**a**) Optimum variations of *w* when $\alpha$ is varied. (**b**) Optimum variations of *r* when $\alpha$ is varied.

In order to satisfy the cross-polarization conversion criteria, a simple I-shape element with two additional arc stubs is used as the metal patch of the unit cell. As shown in Fig. 2a, the metal patch is rotated by $\beta$ angle (with two values of ${45^ \circ }$ and ${135^ \circ }$) with respect to the x-axis to exhibit anisotropic behavior. The unit cell dimension of the proposed single layer element is 10 mm$\times$10 mm. The polarization rotation purity of the element and the phase range of the reflection wave from the element in orthogonal polarization are two determinative factors for selecting the unit cell dimension. The proposed element and its mirror have enough freedom to make a similar phase shift over the cross-polarization wave in the entire band. As can be observed in Fig. 2a, the I-shaped metal patch is placed on a single layer dielectric whose thickness and dielectric constant are 0.79 mm and 2.33, respectively. A gap with a given thickness of 3.8 mm is considered between the ground plane and the dielectric to further enhance the bandwidth. The construction steps of the metal patch element are schematically illustrated in Fig. 2b. The primitive sketch of the I-shape metal patch is constructed by a ring and a strip patch. Two symmetric arc-shaped sections are omitted from the main patch to construct an I-shape metal patch. Finally, the obtained patch is rotated by $\beta$ angle. As shown in Fig. 2b, $\alpha$ is assigned to the opening angle subtended by these two arc-shaped sections. The phase of the reflected cross-polarization wave changes dominantly by varying the $\alpha$ parameter as the first degree of freedom (FDF). It can simply be shown that the phase behavior of the element cannot meet the expectations and the element has no similar phase behavior in a wide frequency range by using only the FDF technology. Therefore, the MDoF technology is adopted to meet the expectations. In doing so, a full parametric study is conducted by the CST software. It is found that three geometrical parameters, *w*, *r*, and $\alpha$, should be varied at the same time to observe wideband behavior for the element in the cross-polarization mode. Figure 3 illustrates the optimum relationship between *w*, *r*, $\alpha$, when $\alpha$ is changed from ${10^ \circ }$ to ${160^ \circ }$ to guarantee the wideband behavior of the unit cell. To rotate the reflected polarization wave by ${90^ \circ }$ with respect to that of illuminated from the feed, the value of the $\beta$ parameter should be selected as either ${45^ \circ }$ or ${135^ \circ }$. This ensures the anisotropic behavior of the element. Simulations show that if $\beta$ is selected equal to ${45^ \circ }$, all combinations of *w*, *r*, $\alpha$ provide only half cycle of the required phase in Ku-band, that is ${180^ \circ }$ in the cross-polarization mode. Therefore, a full cycle of ${360^ \circ }$ for the reflecting phase, which is required to implement a completely equiphase aperture in the cross-polarization mode, can easily be realized by selecting $\beta = {45^ \circ }$ and ${135^ \circ }$.

**Figure 4:**
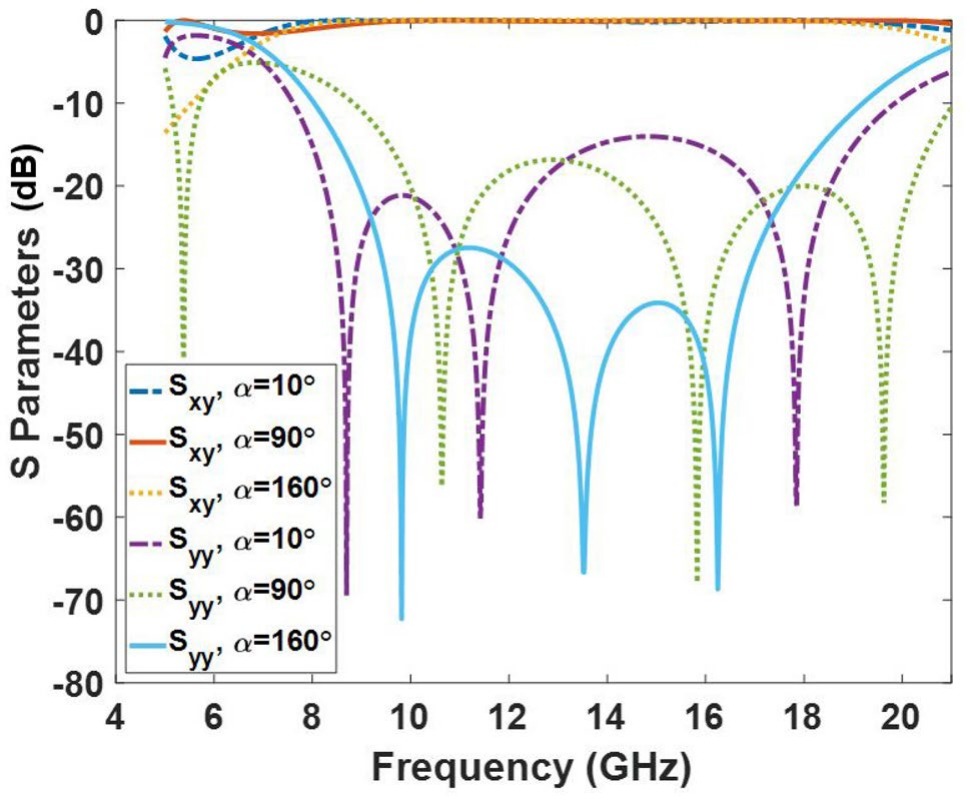
Co- and Cross-polarization conversion coefficients of the element for various values of $\alpha$. In this plot $\beta = {45^ \circ }$. The parameters *w* and *r* are determined from Fig. 3. The quantities ${S_{xy}}$ and ${S_{yy}}$ are the reflection coefficients of the x- and y-polarized waves under the illumination of the y-polarized wave, respectively.

In light of the above discussion, when the $\alpha$ parameter changes, other geometrical parameters (*w* and *r*) should be selected from Fig. 3. The cross-polarization conversion behavior of the I-shaped element for various values of $\alpha$ is depicted in Fig. 4 when the y-polarized wave is assumed as the impinging wave. It is depicted that the ${S_{yy}}$ is smaller than -15 dB from 10 GHz to 18 GHz for all values of $\alpha$ indicating good polarization conversion performance. Three resonance frequencies are observed in the frequency band which vary with the $\alpha$ parameter. Undoubtedly, the intense induced current distribution is formed on the metallic patch at these resonance frequencies.

**Figure 5:**
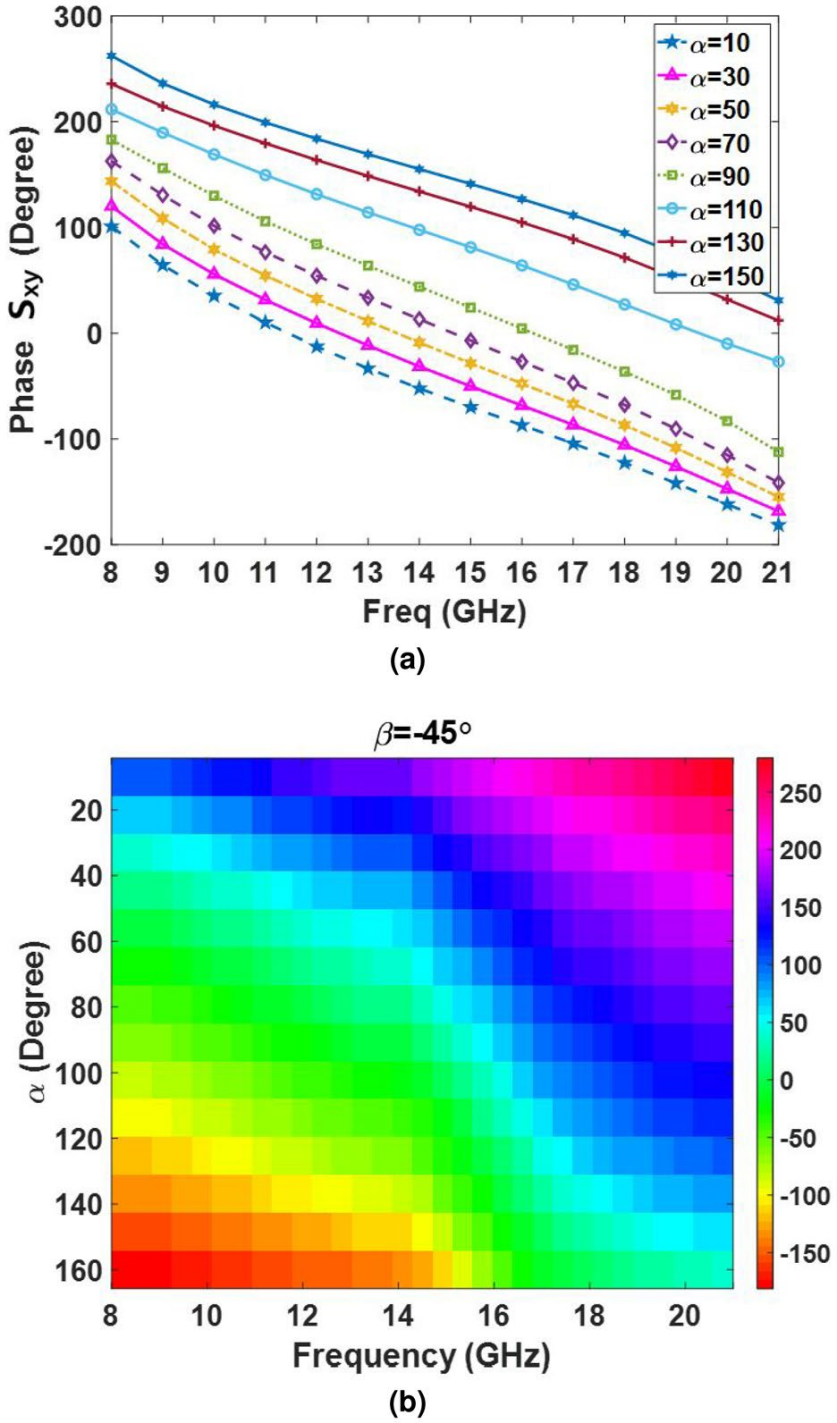
(**a**) and (**b**) The cross-polarized reflection coefficients versus the frequency for different values of $\alpha$ angles.

**Figure 6:**
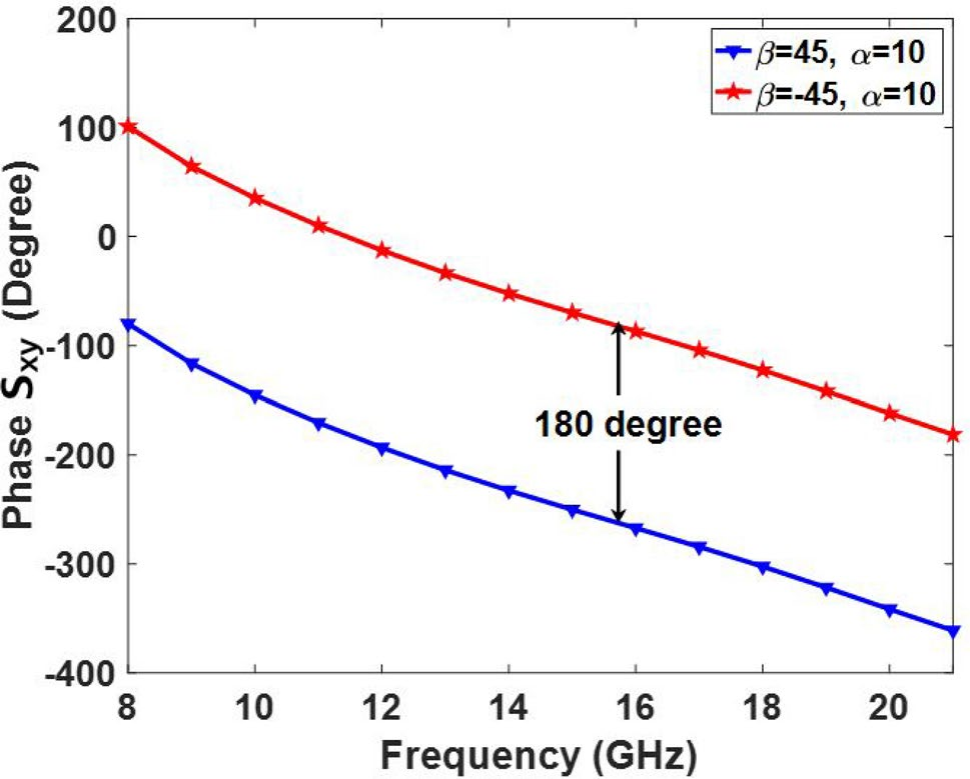
Reflection phase of ${S_{xy}}$ for $\beta = {45^ \circ }, - {45^ \circ }$. The $\alpha$ parameter is considered to be ${10^ \circ }$.

**Figure 7:**
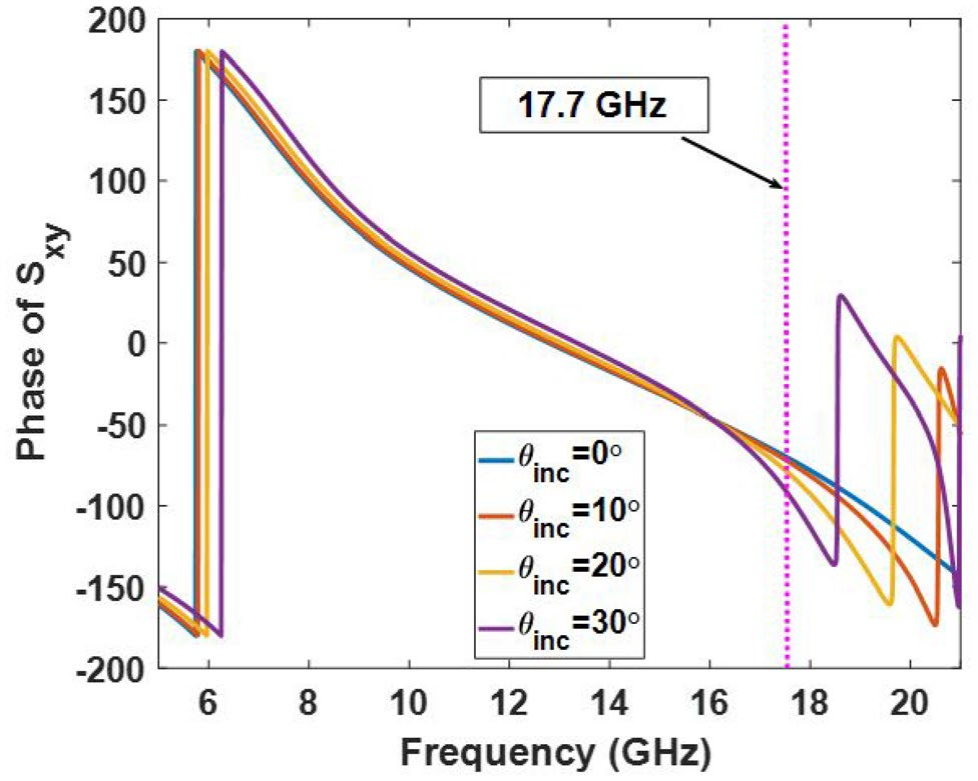
Reflection phase of ${S_{xy}}$ for various incident angles of the wave illuminated from the feed.

The reflection phase of the cross-polarization wave with various values of $\alpha$ are shown in Fig. 5 for $\beta = -{45^ \circ }$. It can be seen from Fig. 5a that the reflection phase of the cross-polarization component has a similar behavior from 8 to 21 GHz; however, the maximum coverage of the phase is limited to ${180^ \circ }$. Because of symmetry, its mirror (with $\beta = {45^ \circ }$) has similar behavior in the entire band which has a ${180^ \circ }$ phase difference as shown in Fig. 6. To complete the discussion, the effect of the incident angle of the wave is also examined over the cross-polarization reflection phase of the element within the band. Figure 7 shows the phase of ${S_{xy}}$ for various values of the angle of incidence. As can be seen in Fig. 7, the reflection phase behavior is nearly independent of the incident angle of the wave from 7 to 18 GHz. Although, the reflecting phase of the cross-polarization component deteriorates beyond 18 GHz, leading to a decrease in the antenna performance. Note that a few elements placed near the edge of the metasurface are subjected to waves with high angles of incidence, so the undesirable effect of this phase degradation is mainly observed in the aperture efficiency and it has a negligible effect on the shape of the reflected beam. This phenomenon is experimentally demonstrated in the measurement results where the gain of the reflected wave dramatically decreases beyond 19 GHz.

Following the guideline described above, an equiphase aperture can be implemented by arranging various elements with independent geometrical parameters. The key factor for selecting the geometrical parameter associated with each element is its required reflection phase which is dictated by generalized Snell’s law for that position. Accordingly, when the required phase distribution is determined by generalized Snell’s law to guide the reflection beam at a given direction, $\alpha$ is determined and, in the next step, *w* and *r* will be determined from Fig. 3.

## Reflecting metasurface design by combining the TFPS method and PCT

Before explaining the TFPS method and how it can be combined with PCT to considerably enhance the frequency bandwidth of the reflecting metasurface, some considerations need to be expressed. We know that anomalous reflection occurs near the interface of the metasurface and can be described by generalized Snell’s law which relates the incidence angle of the wave, anomalous reflection direction, and the local phase gradient to each other^28^. In other words, for a beam reflected at a given direction, the desired phase distribution on the reflecting metasurface is dictated by the generalized Snell’s law.

In general, all phases associated with all unit cells can be added or subtracted by a given constant value, named the constant reference phase. The value of the constant reference phase is not important in the single-frequency synthesis approach. However, in the dual- or multi-frequency synthesis approach, the use of different constant reference phases results in great variations in the electromagnetic features of the metasurface such as the realized gain. Therefore, the determination process of these constant reference phases can be performed intelligently to enhance the bandwidth. The constant reference phases can effectively be determined during an optimization process. To do this, a cost function is defined so that the phase error at two or more separate frequencies is minimized for all elements. This ensures that the final optimized phase distribution, which would be provided by the elements, has small variations when the frequency changes. We employ this approach at three frequencies in this work which is named TFPS. In this regard, the required phase, phase error, and the cost function are all represented in (1), (2), and (3).

1 $$\begin{aligned}&{\phi _{mn,r}}\left( {{f_i},\Delta {\phi _i}} \right) = {k_i}.\left( {\left| {{{{\bar{r}}}_{mn}} - {{{\bar{r}}}_f}} \right| - {{{\hat{u}}}_0}.\,{{{\bar{r}}}_{mn}}} \right) + \Delta {\phi _i} \end{aligned}$$

2 $$\begin{aligned}&P{E_{mn}}\left( {{f_i},\Delta {\phi _i}} \right) = \left| {{\phi _{mn,a}}\left( {{f_i},\Delta {\phi _i}} \right) - {\phi _{mn,r}}\left( {{f_i},\Delta {\phi _i}} \right) } \right| \end{aligned}$$

3 $$\begin{aligned}&CF\left( {{f_i},\Delta {\phi _i}} \right) = \sum \limits _{m,n} {\left[ {\sum \limits _i {{w_{i,mn}}P{E_{mn}}\left( {{f_i},\Delta {\phi _i}} \right) } } \right] } \end{aligned}$$

In the above equations, the *i* parameters are selected as 1, 2, 3, for TFPS. The quantities $${\phi _{mn,r}}$$ and $${\phi _{mn,a}}$$ are the required and achievable phases for the element *mn*, respectively. $$P{E_{mn}}$$ denotes the phase error of the element *mn*. The weighting coefficients $${{w_{i,mn}}}$$ are determined based on the illumination intensity over the elements on the metasurface. These coefficients are defined based on the point source’s characteristics and its relative position with respect to the metasurface. These coefficients are defined for every element at a specific frequency. Herein, particle swarm optimization (PSO) as a global search algorithm is employed to minimize the cost function. The role of the element and its phase-shifting behavior versus frequency are observed in (2) which can considerably decrease the phase error if a wideband unit cell is designed for this purpose. Investigations show that the choice of three frequencies, $${f_1} $$, $${f_2}$$, and $${f_3}$$, can directly affect the metasurface bandwidth and, hence, the best choice is made after several iterations of the algorithm for various values of frequencies in the band. To accelerate this process, we used the 2D-FFT algorithm to characterize the radiated beam for every phase distribution. That is, for all three frequencies, the optimum phase distribution for the aperture is obtained by the proposed algorithm; afterward, the radiated beam is calculated by 2D-FFT and the 1-dB gain bandwidth is immediately evaluated. It is found that the best performance of the TFPS technique is achieved by choosing $${f_1} $$= 14.5 GHz, $${f_2}$$= 17 GHz, and $${f_3}$$= 19 GHz, and the corresponding optimum constant reference phases $$\Delta {\phi _1}$$, $$\Delta {\phi _2}$$, and $$\Delta {\phi _3}$$ are obtained equal to $${47^ \circ }$$, $${223^ \circ }$$, and $${80^ \circ }$$, associated with $${f_1}$$, $${f_2}$$, and $${f_3}$$, respectively, all extracted from the PSO algorithm.

## Feed horn description

**Figure 8:**
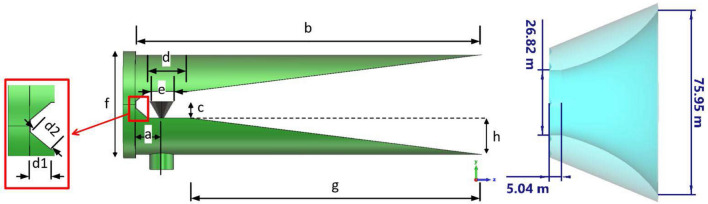
Antenna feed along with its wideband adapter. The conical waveguide section of the horn is not shown in this figure to better represent the transition section.

**Figure 9:**
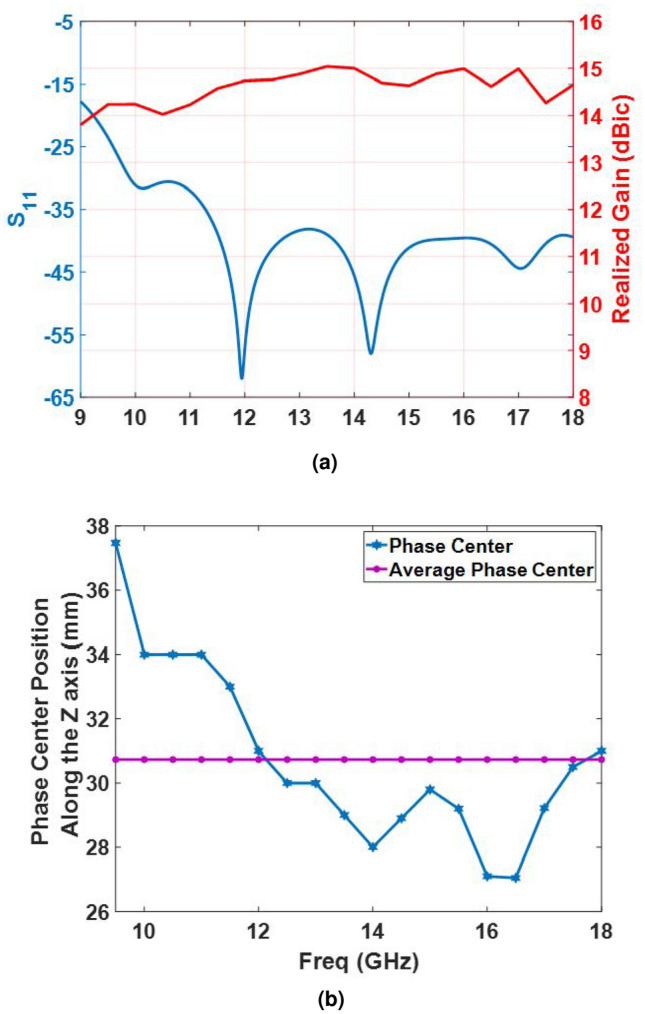
(**a**) Realized gain along with the ${S_{11}}$ parameter of the horn antenna. (**b**) Phase center variations versus frequency and its average. (The ${S_{11}}$ parameter is shown in dB scale).

In a practical case, a point source should be used to illuminate the metasurface. The best choice is to utilize a horn antenna whose phase center has minimum variations versus frequency. This ensures that the feed has a minimum undesirable effect on the metasurface and, hence, the best performance of the metasurface is achievable. Since the traditional horn antennas experience great variations in their phase center positions when the frequency varies, a tangential profiled smooth-wall horn is designed to feed the metasurface so that its phase center variation is negligible from 10 to 18 GHz. A wideband coaxial-to-waveguide transition is also designed to excite the horn. The optimum dimension of the horn and its transition section are presented in Fig. 8 and Table 1. The electromagnetic characteristics of the horn including reflection co-efficient, realized gain, and the phase center position with respect to the front of the horn aperture are all depicted in Fig. 9. Evidently, antenna gain varies from 13.8 to 15.1 dBi in the frequency band of 9–18 GHz. In addition, the variations of the phase center position are limited to 4 mm when the frequency changes from 12 to 18 GHz, which is an excellent achievement for illuminating the metasurface. This ultimately leads to the assumption that the focal-to-diameter ratio (f/D) is nearly fixed and consequently, the contribution of phase variation on the aperture of the metasurface imposed by the point source (or horn) would be small when the frequency is changed.

**Table 1:** Optimum values of design parameters of the wideband coaxial-to-waveguide transition.

Parameter name	a	b	c	d	e	f	g	h	d1	d2
Value (mm)	6.5	86.5	4.1	10.3	6	26.82	73.2	9.2	3	3.9

## Fabricated prototype and measured results

**Figure 10:**
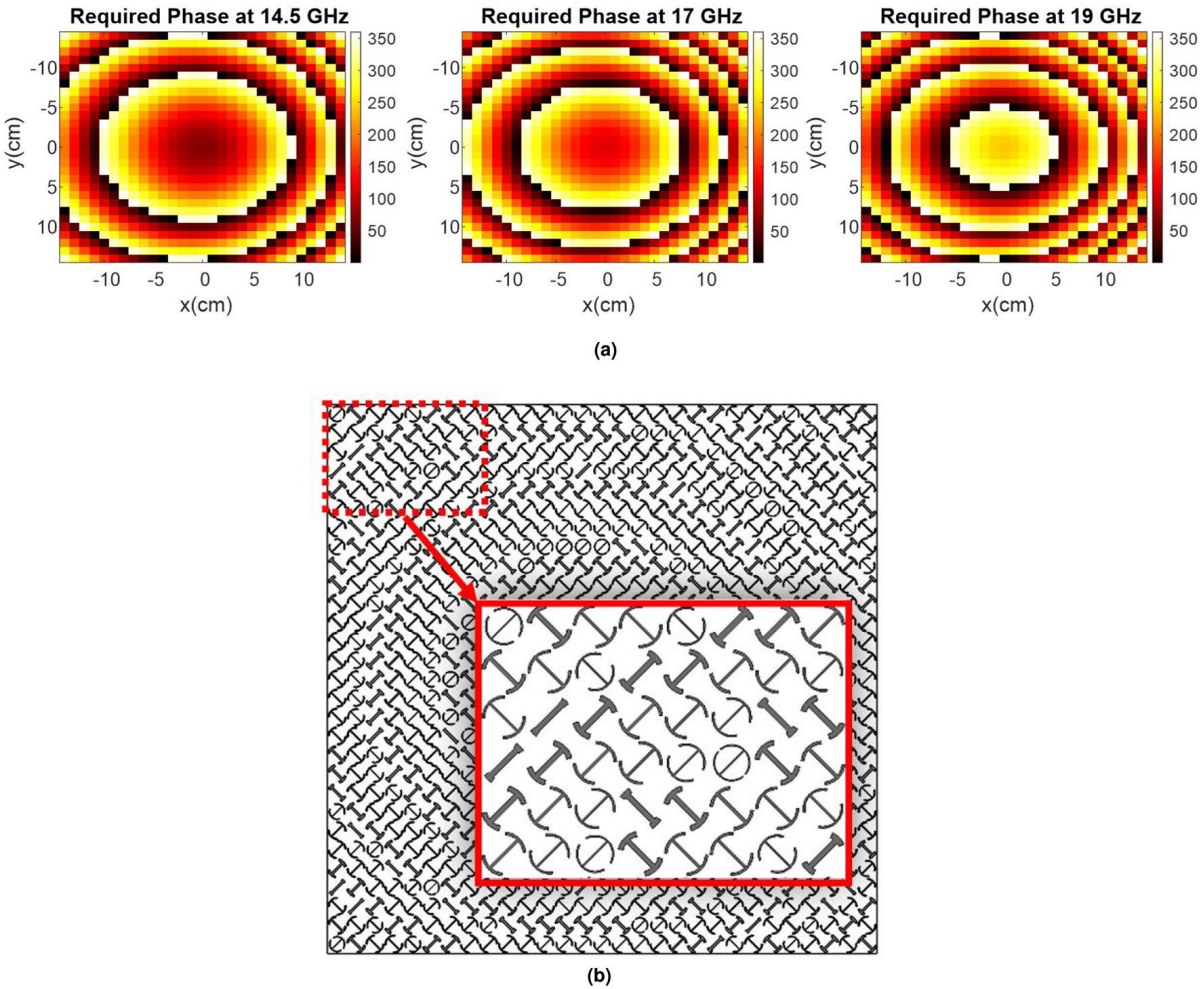
(**a**) Required reflection phase at three frequencies of $${f_1} $$= 14.5 GHz, $${f_2}$$= 17 GHz, and $${f_3}$$= 19 GHz. (**b**) Arrangement of the elements obtained by combining the TFPS method and PCT.

To depict the effectiveness of combining the two aforementioned broadening techniques at system and element design levels, a representative metasurface is designed, simulated, and fabricated in the Ku-band. In doing so, a 29 cm$$ \times $$29 cm aperture including 841 phasing elements is constructed and illuminated by a feeding horn whose phase center is located at (84mm, 0, 232mm) and is pointed to the center of the metasurface aperture. The feed pattern is modeled by $${\cos ^q}\theta $$ function with $$q=6.5$$ ($$f/D = 0.85$$). In order to decrease the feed blockage effect, the feed horn antenna is offset $${20^ \circ }$$ from the boresight direction, and the reflected main beam is designed at $${20^ \circ }$$. Following the design process mentioned above, the phase map at three frequencies of $${f_1} $$= 14.5 GHz, $${f_2}$$= 17 GHz, and $${f_3}$$= 19 GHz, and the final arrangement of the array elements are obtained and demonstrated in Fig. 10a and b.

**Figure 11:**
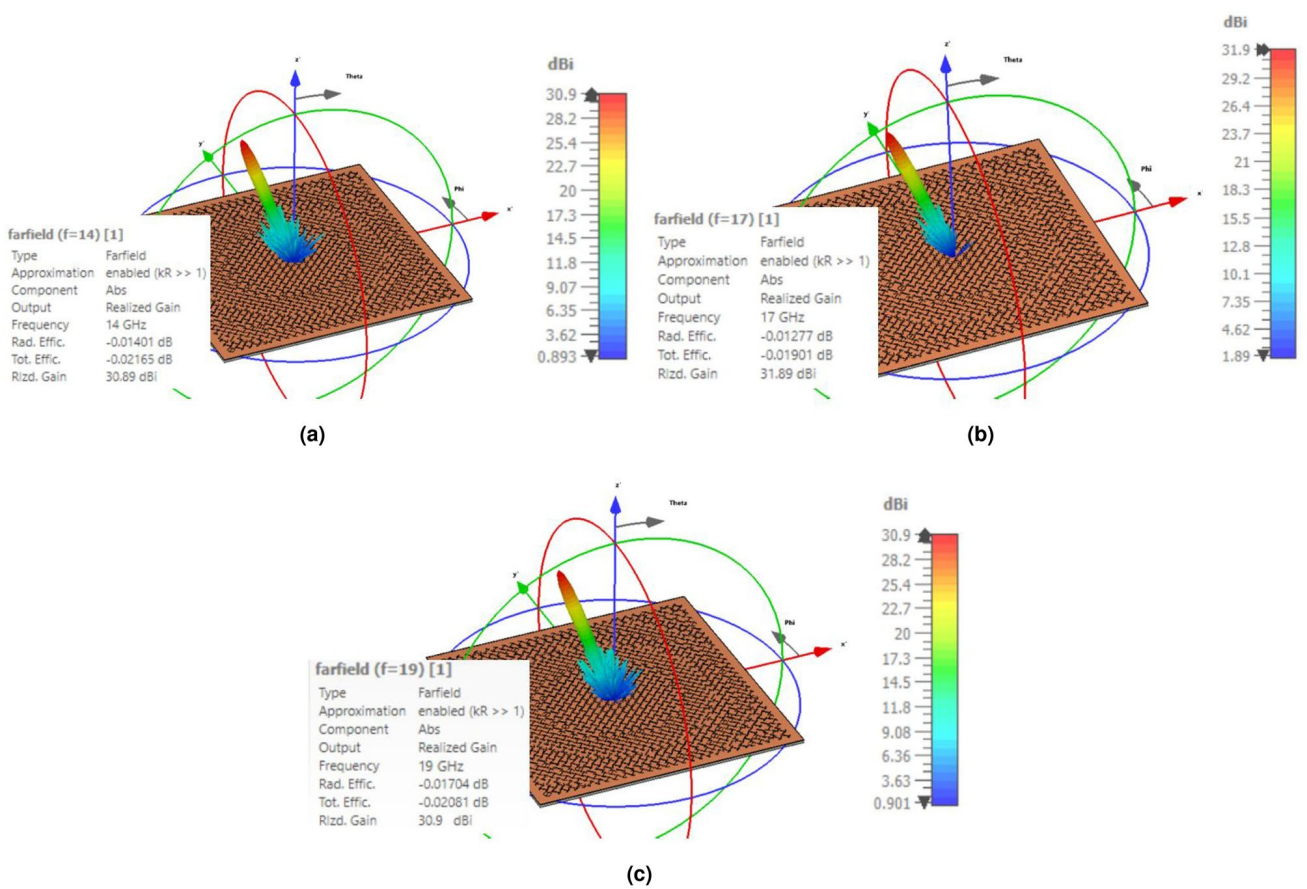
3D demonstration of the pattern designed by the PRT+TFPS method. (**a**) f=14 GHz. (**b**) f=17 GHz. (**c**) 19 GHz.

**Figure 12:**
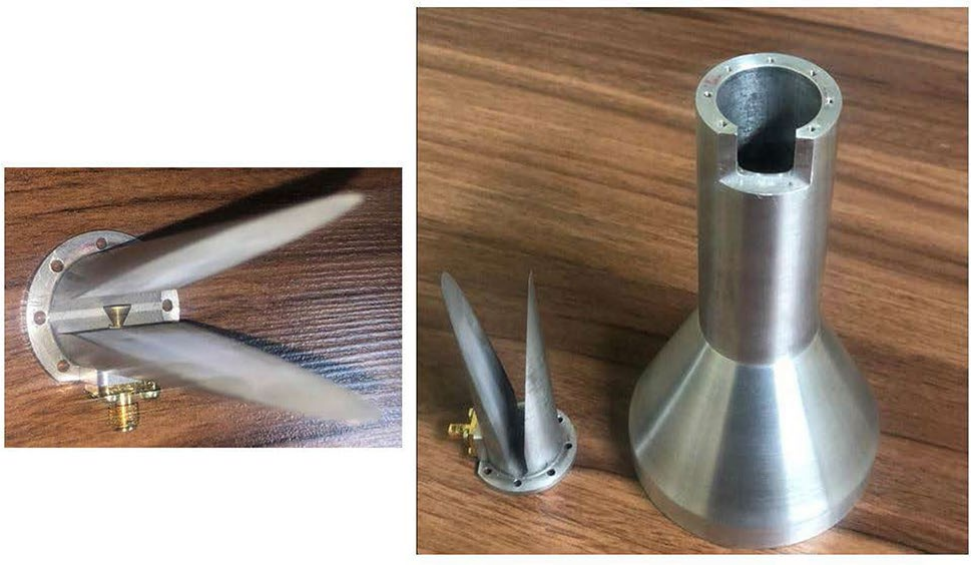
Fabricated horn antenna along with its wideband coaxial to waveguide transition.

**Figure 13:**
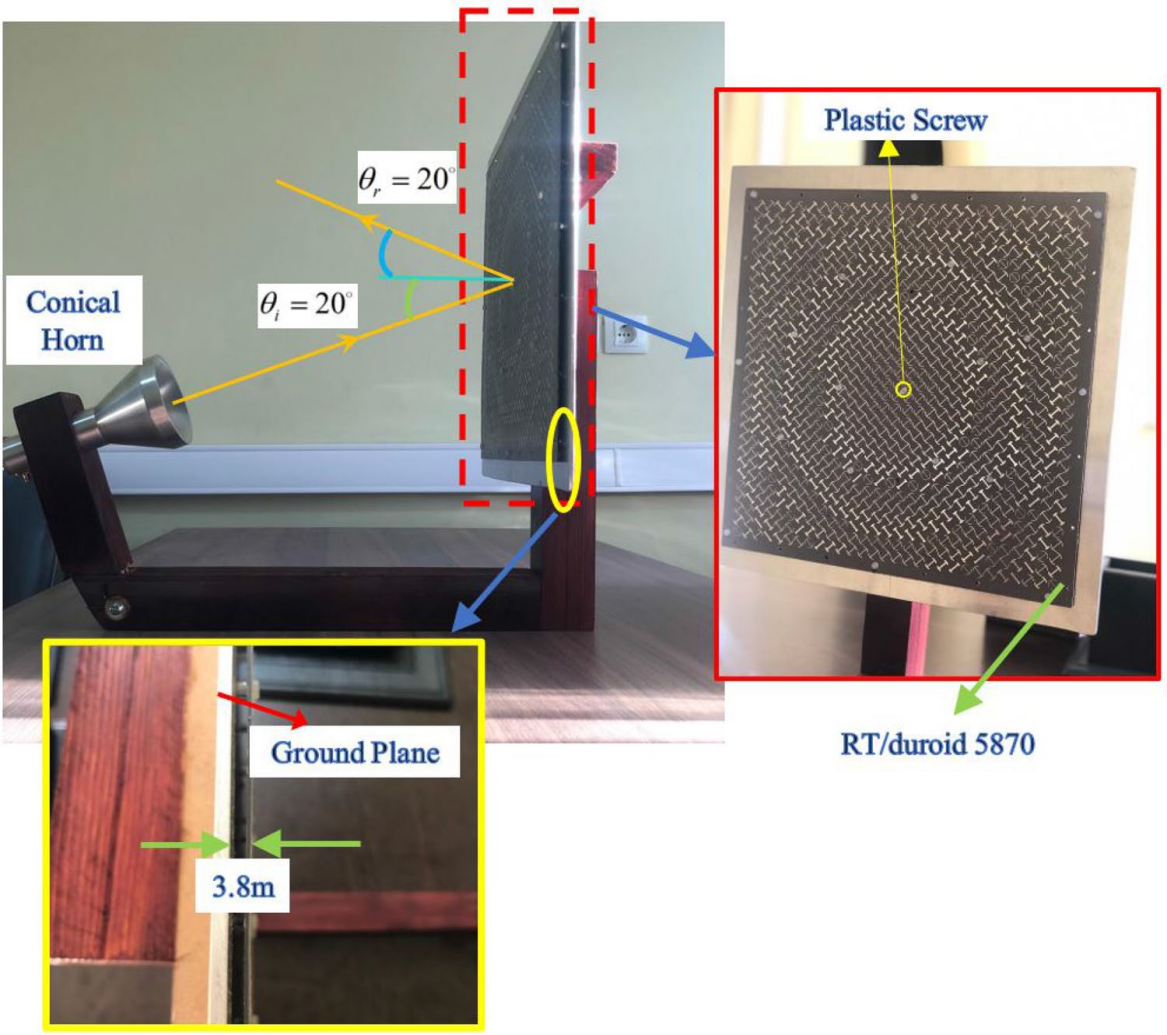
Fabrication of a prototype of metasurface and its position with respect to the horn antenna as the feeding system.

The metasurface along with the feed horn is simulated in the CST software with the time domain (TD) solver. The simulation is carried out on a PC using an Intel Core i7 3.8 GHz processor with 64 GB RAM and 1 TB SSD. Lossy copper (copper, annealed) is used as a metal. In addition, the open (add space) option is selected for the boundary condition in all directions and a waveguide port is used as the feeding. The three-dimensional (3D) demonstration of the patterns of the metasurface designed by the PRT+TFPS method is depicted in Fig. 11 at three frequencies of 14, 17, and 19 GHz. Based on Fig. 11, the general shape of the 3D patterns is acceptable, especially in the back-lobe region, indicating the correct position of the phase center of the horn with respect to the metasurface and minimum phase error at these three frequencies. Additionally, the main beams are appropriately aligned to the predefined direction, showing that the progressive phase on the metasurface is calculated accurately in the entire band based on the TFPS method. Furthermore, the perfect beam collimation ensures the high aperture efficiency for the metasurface. A prototype of the designed conical feed horn is shown in Fig. 12 which is fabricated by a machine controlled by computer numerically (CNC) with high accuracy. The waveguide and flare section of the antenna are fabricated as one unit, and the transition section is fabricated separately and assembled with some screws at the bottom. The fabricated metasurface is shown in Fig. 13. It is composed of a single copper cladding printed circuit board (PCB) using RT$/$duroid 5870 laminate and an aluminum metal plate as the ground. In order to keep the distance between the ground plate and dielectric layer, some spacers made up of plexiglass are placed between the two layers and are fixed by plastic screws (see the inset of Fig. 13). As can be shown in Fig. 13, a wooden stand is fabricated to hold the horn antenna in front of the metasurface with ${20^ \circ }$ offset.

**Figure 14:**
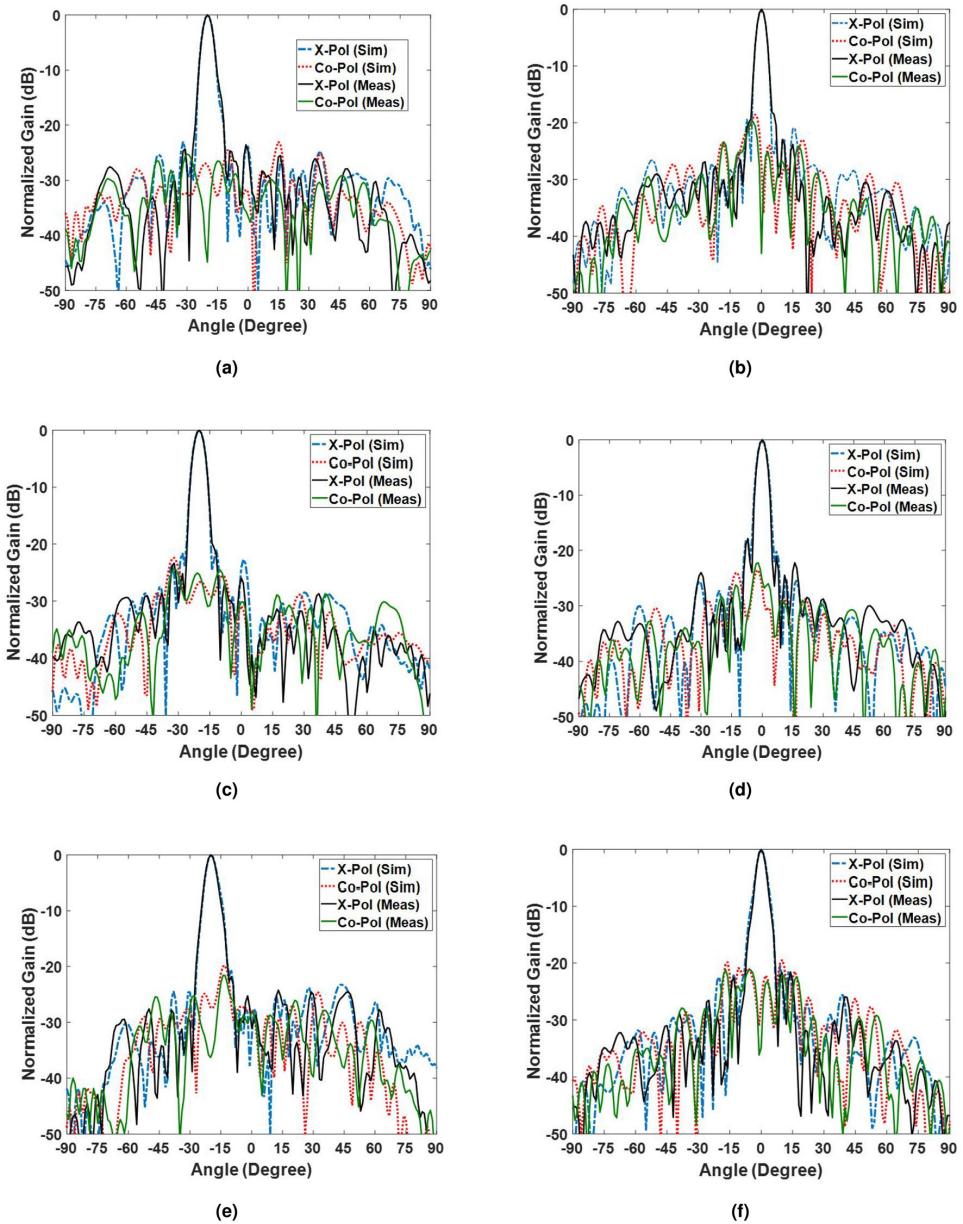
Comparison of the simulation and measurement results. (**a**) E-plane at 14 GHz, (**b**) H-plane at 14 GHz, (**c**) E-plane at 17 GHz, (**d**) H-plane at 17 GHz. (**e**) E-plane at 19 GHz. (**f**) H-plane at 19 GHz.

The simulation and measurement results for the co- and cross-polarization patterns on the E- and H-plane are presented in Fig. 14 at two extreme frequencies (i.e, 14 and 19 GHz) when the x-polarized wave is illuminated by the horn. It is clear that the sidelobe levels are below $-18$ dB for all of the patterns in the E-plane and below $-20$ dB in the H-plane thanks to the use of a completely equiphase aperture. In addition, the cross-polarization levels are all below $-18$ dB, which are acceptable results.

## Method

The measurement of the electromagnetic characteristics of the metasurface is carried out in two steps. First, the S-parameter and the pattern of the horn antenna are measured by a keysight vector network analyzer ENA E5080B series operating up to 20 GHz. Second, the radiation pattern of the radiated beams from the metasurface is measured by this network. In order to measure the realized gain, the comparison technique is adopted by using two standard horn antennas working in Ku- and K-bands.

## Discussion

**Figure 15:**
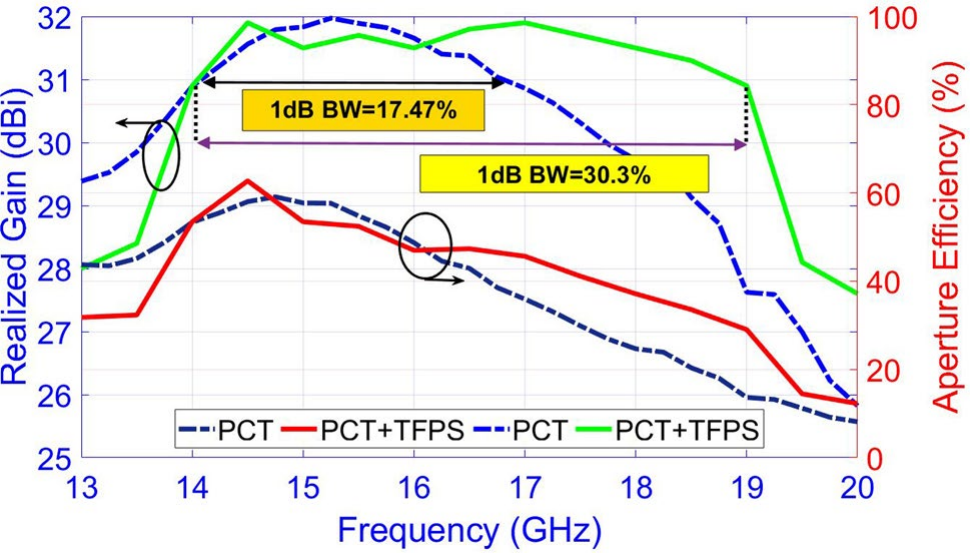
Comparisons of the bandwidth and aperture efficiency of the two metasurfaces developed by the PRT and the PCT+TFPS methods. Note that the measurement results of the metasurface developed by PCT+TFPS are compared with the simulation results of the metasurface developed by PCT.

In order to benchmark the usefulness of the proposed broadening technique which is based on combining the TFPS method and PCT, a comparison is made between the frequency bandwidth of two metasurfaces; the first one is designed by using only PCT, while the second one is designed using the PCT+ TFPS method. The bandwidth and aperture frequency of the two configurations are presented in Fig. 15. As can be seen in Fig. 15, the 1-dB gain bandwidth of the first and second metasurfaces is 17.47% (14.1-16.8 GHz) and 30.3% (14-19 GHz), respectively. This result verifies the advantage of combining the PCT and TFPS method where an increment of nearly 13% is obtained in the bandwidth. The maximum aperture efficiency for the first and second metasurface is 60% and 63.62%, respectively, which occur at 14.5 GHz in both cases. As expected, three peaks are observed in the gain versus frequency plot. These peaks occur at 14.5 GHz, 17 GHz, and 19 GHz which are exactly the same as those defined as the designed frequency in the TFPS method. As stated above, at the system design level, several optimizations are accomplished to find the best three frequencies leading to the maximum 1-dB gain bandwidth. That is, the choice of three frequencies in $CF\left( {{f_i},\Delta {\phi _i}} \right)$ influences how the gain level varies in the entire band and hence changes the 1-dB gain bandwidth.

In order to better highlight the effectiveness of the broadband technique introduced in this work, a comparison is made between several works presented in the literature (Table 2). The comparisons show that the combination of the PCT and TFPS method improves the 1-dB gain bandwidth, low side lobe level, low cross-polarization level, and aperture efficiency concurrently.

**Table 2:** The comparison between this work and previous works.

Reference	Ref.^20^	Ref.^29^	Ref.^30^	Ref.^31^	Ref.^19^	Ref.^32^	This work
Central frequency [GHz]	20	9	10	10	10	8	15
Polarization	LP and CP	LP	LP	LP	LP	LP	LP
Maximum aperture efficiency [%]	54.4	17.6	NR	48.2	20	55	64
Number of metal layers	1	1	3	4	2	1	1
Element dimension [mm]	7.5	7	6.5	15	6	18	10
Aperture dimension [mm]	26${\lambda _0}$	9${\lambda _0}$	8.4${\lambda _0}$	6.5${\lambda _0}$	9.6${\lambda _0}$	8${\lambda _0}$	${14.5}{\lambda _0}$
Maximum Gain [dBi]	35.6	23	23	24	24.2	26.5	31.9
Side lobe level [dB]	$<-15$	$-15$	$-10$	$-17$	$-13$	$- {9^\dag }$, $- {13^{\dag \dag }}$	$- {18}^\dag$, $- {22^{\dag \dag }}$
F/D	0.92	1	0.87	1.08	1	0.75	0.85
Bandwidth [%]	18.5	25	40${^*}$	18.6	24	35	30.3
Broadband techniques	PCT	1-bit	TTD	PCT	PCT	TDFE	PRT+TFPS

The asterisk (${^*}$) in the bandwidth row denotes the 4-dB bandwidth. Other references report 1-dB gain bandwidth. In addition, TDFE stands for two degrees of freedom elements, and superscripts ${^\dag }$ and ${^{\dag \dag }}$ denote the initial and end frequencies of the band. ’NR’ stands for the not-reported data.
